# *Auxarthron alboluteum* related to non-dermatophytic toenail infection in Kurdistan region, Iraq: A case report

**DOI:** 10.1016/j.mmcr.2019.10.006

**Published:** 2019-10-31

**Authors:** Sazan Jamal Gharib, Samir Khalaf Abdullah, Malcolm D. Richardson

**Affiliations:** a Medical Laboratory Department, Sulaimani Technical Institute, Poly Technique University, Kurdistan Region, Iraq; b Biology Department, Faculty of Science, University of Zakho, Kurdistan Region, Iraq; c Medical Laboratory Technics Department, Alnoor University College, Nineva, Iraq; d NHS Mycology Reference Centre, Wythenshawe Hospital, Manchester University, UK

**Keywords:** *Auxarthron alboluteum*, Onychomycosis, Toenail infection, ITS region, Kurdistan region, Iraq

## Abstract

We report a rare case of non-dermatophytic onychomycosis of the big toenail caused by *Auxarthron alboluteum* in a 63-years old Iraqi female with a history of diabetes. The big toenail showed distal subungual onychomycosis with extensive yellow-white discoloration. Identification of the causative agent was confirmed by morphological and microscopical characteristics in culture and analysis of ITS-rDNA region. To the best of our knowledge, the isolated *Auxarthron alboluteum* reported here is a new etiologic species of nail infection in Iraq and this is the first case of its kind to be reported in the world.

## 1 Introduction

Fungal nail infection (tinea unguium) is a term strictly used for nails infection due to dermatophytic genera *Microsporum, Trichophyton, Epidermophyton, Arthroderma, Nannizzia*, *Lophophyton* and *Guarromyces* [1]. Onychomychosis is a chronic fungal disease and is a general term described for dermatophytic and nondermatophytic nail infections. Among the most frequently nondermatophytic fungi encountered as causal agents in nail infections are species in the genera *Acremonium, Alternaria, Aspergillus, Candida, Fusarium Geotrichum, Scopulariopsis* and *Scytalidium* [2]. Several studies demonstrated that the most common etiological agents among patients with nail infections were dermatophyte, followed by moulds and yeasts [1,3,4]. Our aim was to identify and report a nondermatophytic onygenalean fungus causing onychomycosis of a toenail of the 63-years old elderly women from Kurdistan region, Iraq.

## 2 Case

**Fig. 1:**
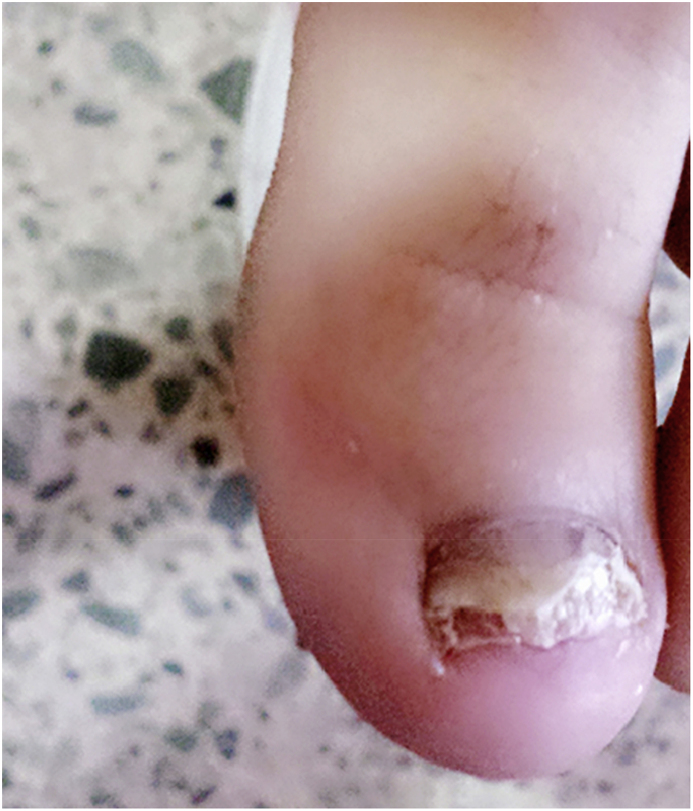
Toenail of the feet of 63 years-old woman showing distal subungual onychomycosis due to *Auxarthron alboluteum* with extensive yellow-white discoloration.

A 63-year-old female diabetic patient, presented in January 2017 (day 0) to the Dermatology out-patient department of Khabat Hospital with a dystrophic great left toenail, showing yellowish white to creamy discoloration (Fig. 1) of 9 months duration (-9 months). There was a history of probable onychomycosis of her left great toenails (not verified by mycological examination) three years previously (-3 years), that was completely cured after treatment with antifungal clotrimazole cream 1% (fugidin cream, SDI) used 3times/day for exactly 2 months. The associated predisposing factor for this case was diabetes mellitus, and at the time of sampling, the patient had undergone diabetic medication. No history of any underlying disease was reported.

**Fig. 2:**
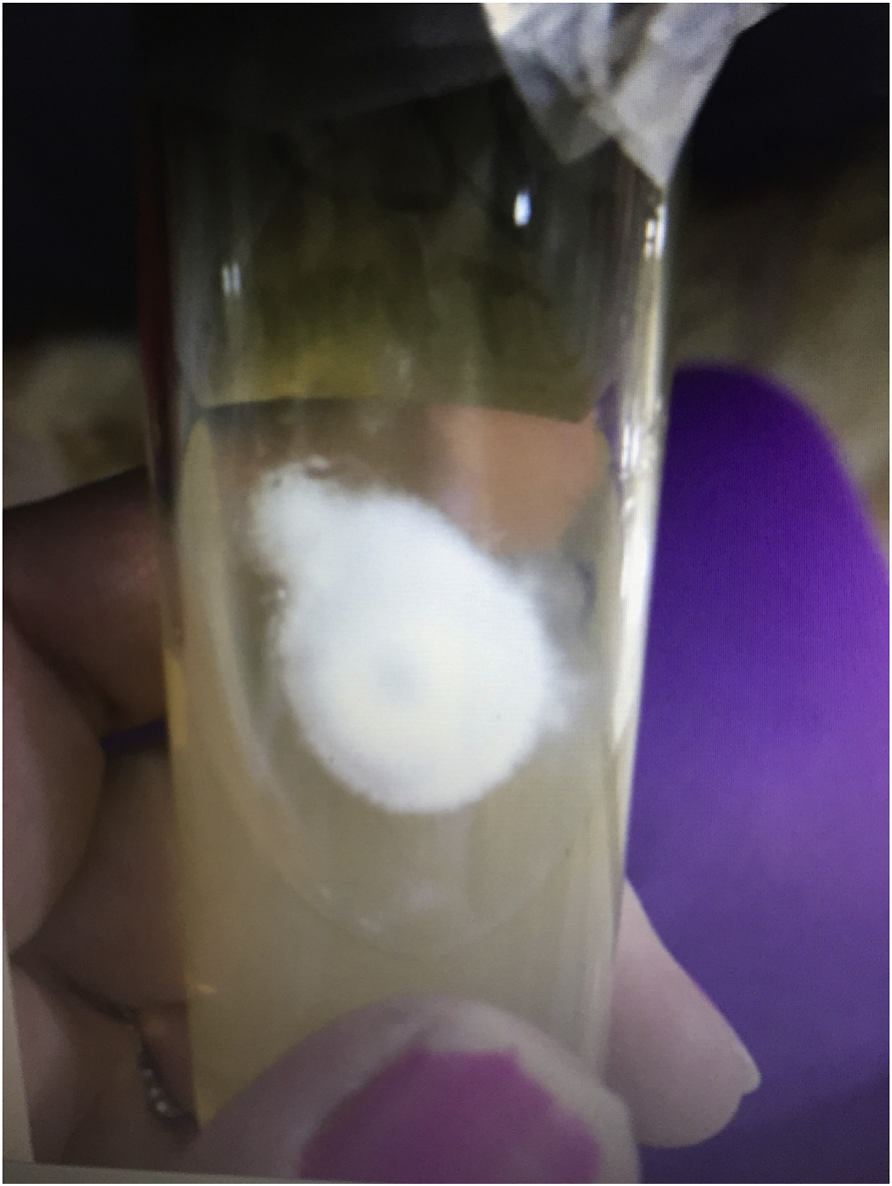
Colony growth of *Auxarthron alboluteum* after three weeks of cultivation at 28 °C on SDA slants.

**Fig. 3:**
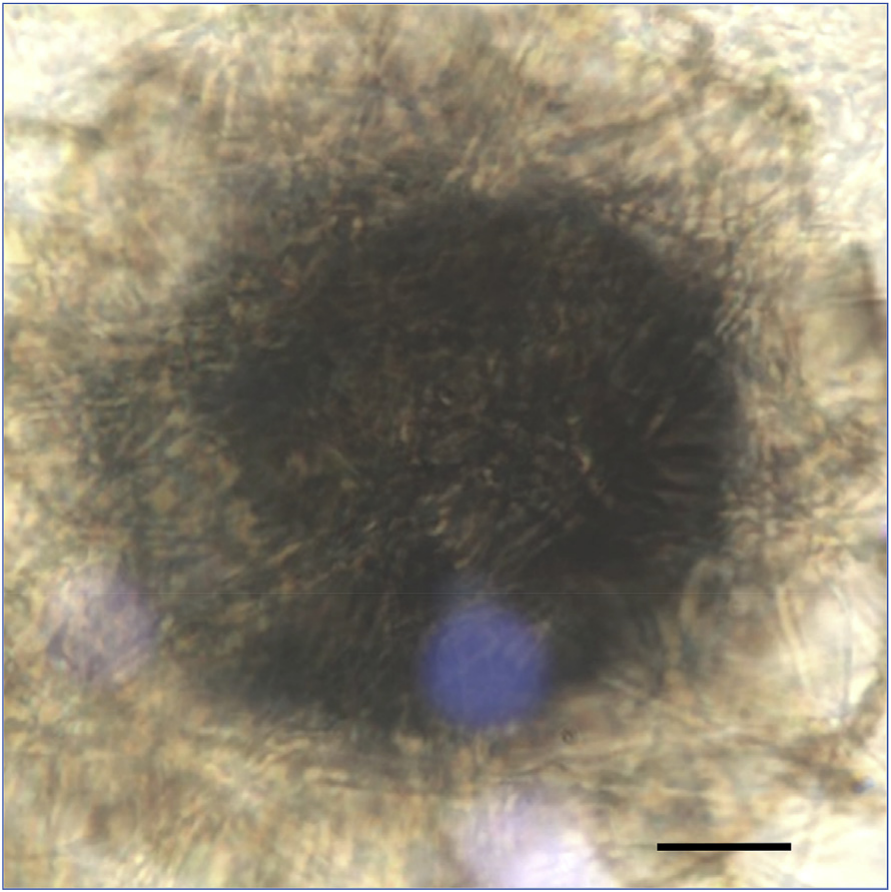
Gymnothecium of *Auxarthron alboluteum*. Bar = 50 μm.

Nail scrapings and clippings from different parts of the affected toenail were collected on day 0 after proper sterilization of the affected area with 70% alcohol and transported in folded dry-square of paper envelope to the mycology research laboratory, Biology Department at Zakho University, for direct microscopic examination and culture processing [5]. On direct KOH examination, septate filaments and arthroconidia were observed. At +3 days Portions of the nail scrapping and clippings were cultured on two sets of Sabouraud's dextrose agar (SDA) medium (CM41; Oxoid, UK): two replicates of SDA plates with antibiotics and cycloheximide [Sigma-Aldrich] (500 mg/l), and one slant of SDA contained antibiotics but without cycloheximide, and incubated at 28 °C for at least 3 weeks. Mycological diagnosis was performed at +21 days. A mesh-like brown globose gymnothecia composed of network of thick-walled hyphae (reticuloperidium) related to onygenalean fungi were grew on both sets of media. No other fungi were detected on both sets of media. The onygenalean fungus was identified as a member of *Auxarthron* based on cultural and microscopic characteristics (Fig. 2, Fig. 3) according to relevant references [[6], [7], [8], [9], [10]]. For accurate identification of the isolated causal agent, DNA sequencing, was performed.

Fungus genomic DNA was extracted from isolated fungus using liquid nitrogen and EZ-10 Spin Column Fungal Genomic DNA Mini-preps Kit, BIO BASIC INC., Canada, and the genomic DNA was analyzed by electrophoresis on 1.5% agarose gel. The ITS region on rDNA was amplified by using universal primers ITS1- (5'-TCCGTAGGTGAACCTGCGG-3') and ITS4 (5'-TCCTCCGCTTATTGATATGC-3') (MWG-Biotech AG, Germany) which yielded a PCR product size of 582bp. The PCR reaction was done in a GenAmp9700 thermal cycler (Perkin-Elmer) under the following conditions: an initial denaturation set up at 94 °C for 5 min. was followed by 35 cycles of denaturation at 94 °C for 1 min., annealing at 58 °C for 1 min. and extension at 72 °C for 1 min., with a final extension step of 72 °C for 10 min.

The sequencing was done at Macrogen company, Korea and the sequence was deposited in GenBank with accession no. MK112622 (ITS). For phylogenetic analysis, sequences of amplified product was retrieved in FASTA format from GenBank. Phylogenetic analyses using the Neighbour-Joining (NJ) method [11] was performed with the MEGA v. X computer program [12]. The phylogenetic tree was constructed using the Kimura two-parameter distance model [13] with the ‘pairwise deletion of gaps option’. The robustness of branches was assessed by bootstrap analysis with 1000 replicates.

At +22 days, the patient was treated with oral itraconazole 200 mg daily for 2 consecutive months. At +82 days, the patient visited the dermatology out-patient department, her toenail was not completely cured, yet, the direct microscopy and culture of nail clippings were negative for fungal elements. At +82 days, oral itraconazole (200 mg/day) in combination with topical fugidin cream (clotrimazole cream 1%) (SDI), twice per day were given for a further 2 months. The patient did not visit the dermatology out-patient department of Khabat hospital after the last therapy, for that reason the patient medical condition cannot be confirmed.

## 3 Discussion

In the present study, we isolated an onygenalean fungus from the toenail of a 63-year old elderly woman that was assigned to the genus *Auxrathron* Orr&Kuehn. *Auxarthron* is characterized by developing globose gymnothecium with reticuloperidium, evanescent asci, with globose or ovoid, finely pitted to echinulate ascospores and a *Malbranchea* anamorph [6,7]. Currently, the genus *Auxarthron* is comprised 16 species according to Refs. [[6], [7], [8], [9], [10],^14^]. Species of *Auxarthron* have been repeatedly isolated from soil [6,14], dung [6,10], river sediments [8], and soil enriched with keratinaceous substances [6].

We confirmed the identification of our isolate to species level as *A.alboluteum* (accession no. MK112622.1) by DNA sequence analysis.

A nucleotide BLAST search with 582bp showed a maximum homology of 99% with *A. alboluteum* of Egyptian origin (MF503899.1), 98% with Greek and Turkish *A. alboluteum* strains (KC253973.1 and KF938454.1) respectively, 95% with *A. alboluteum* from China (KP216931.1) and 94% accordance with several other strains from USA and Canada.

**Fig. 4:**
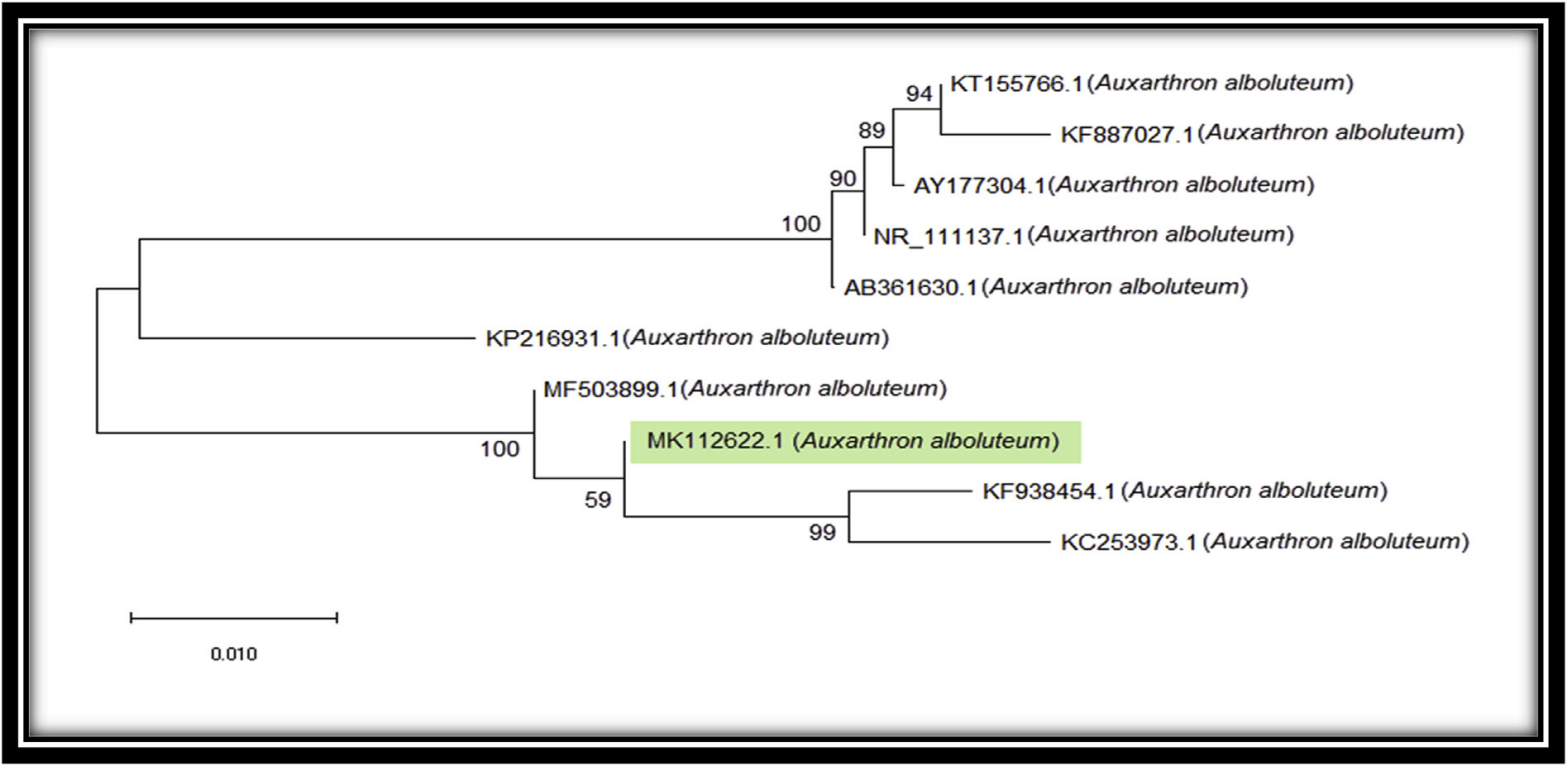
Neighbour-joining (NJ) tree of *Auxarthron alboluteum* based on nucleotidesequence of ITS-rDNA.

The phylogenetic tree based on analysis of ITS rDNA sequences in Fig. 4 evidently designated that our fungal isolate (accession no. MK112622.1) clustered with the accessions (KF938454.1, KC253973.1 and MF503899.1) in a common ancestor with the supporting of 59, 99 and 100 bootstrap. The highest bootstrap of our isolate was revealed with the accession MF503899.1. The comparison between our isolate and the ITS reference sequences that were obtained from the NCBI database confirmed the identification of our isolates as *A. alboluteum*.

*Auxarthron alboletum* has been reported in several occasions as a soil-borne fungus [7,15]. Our report is the first cause of onychomycosis with *A. alboluteum* as a causal agent.

Onychomycosis due to *Auxarthron* species was rarely encountered. There were few reports caused by *Auxarthron*. The first proven case was due to *A.ostraviense* isolated from the fingernails of 26-year old male with psoriasis [9]. Other reports demonstrated *A.umbrinum* as causative agent of onychomycosis on the right great toenail of 36-year old male,54-year old man and a 29-year old woman. All cases were reported from the Czech Republic [9].

Our knowledge on incidence and causal agents of nails infection in Iraq is poorly known, perhaps may be due to a lack of research. A high incidence in tinea ungium (34.9%) was reported in 1999 in Basrah province [16], whereas, in another study, a lower incidence (6.7%) was reported in 2002 in patients with onychomycosis from the same city [17]. From the later study, dermatophytes were responsible for 45.5% of onychomycosis, whereas, yeasts accounted for 54.5% of cases. Etiologic agents of onychomycosis due to dermatophytes were *Epidermophyton floccosum, Trichophyton verrucosum* and *T.violaceum*. Yeasts infections of nails caused by *Candida albicans, C.famata, C.parapsilosis* and *Geotrichum candidum* [17]. A case of white superficial onychomycosis was related to non-dermatophyte *Cladosporium* spp. in a patient from Baghdad [18]. In a recent study, 12% of onychomycosis was related to *Malassezia furfur* infection in patients from Baghdad [19].

In conclusion, to the best of our knowledge, we report the first case of onychomycosis of a toenail of a diabetic elderly women caused by *A. alboluteum*.Identification of the causal agent was confirmed by molecular methods. Ageing and diabetic mellitus are likely to be a predisposing factor in our reported case. The study provides further information's on etiological agents of onychomycosis in Iraq.

## Ethical Form

Please note that this journal requires full disclosure of all sources of funding and potential conflicts of interest. The journal also requires a declaration that the author(s) have obtained written and signed consent to publish the case report from the patient or legal guardian(s).

The statements on funding, conflict of interest and consent need to be submitted via our Ethical Form that can be downloaded from the submission site www.ees.elsevier.com/mmcr. **Please note that your manuscript will not be considered for publication until the signed Ethical Form has been received.**

## Declaration of competing interest

There are none.
